# Kinetics of Blood–Brain Barrier Transport of Monoclonal Antibodies Targeting the Insulin Receptor and the Transferrin Receptor

**DOI:** 10.3390/ph15010003

**Published:** 2021-12-21

**Authors:** William M. Pardridge

**Affiliations:** Department of Medicine, UCLA, Los Angeles, CA 90095, USA; wpardrid@ucla.edu

**Keywords:** blood–brain barrier, brain drug delivery, monoclonal antibody, transferrin receptor, insulin receptor, mathematical model

## Abstract

Biologic drugs are large molecule pharmaceuticals that do not cross the blood–brain barrier (BBB), which is formed by the brain capillary endothelium. Biologics can be re-engineered for BBB transport as IgG fusion proteins, where the IgG domain is a monoclonal antibody (MAb) that targets an endogenous BBB transporter, such as the insulin receptor (IR) or transferrin receptor (TfR). The IR and TfR at the BBB transport the receptor-specific MAb in parallel with the transport of the endogenous ligand, insulin or transferrin. The kinetics of BBB transport of insulin or transferrin, or an IRMAb or TfRMAb, can be quantified with separate mathematical models. Mathematical models to estimate the half-time of receptor endocytosis, MAb or ligand exocytosis into brain extracellular space, or receptor recycling back to the endothelial luminal membrane were fit to the brain uptake of a TfRMAb or a IRMAb fusion protein in the Rhesus monkey. Model fits to the data also allow for estimates of the rates of association of the MAb in plasma with the IR or TfR that is embedded within the endothelial luminal membrane in vivo. The parameters generated from the model fits can be used to estimate the brain concentration profile of the MAb over time, and this brain exposure is shown to be a function of the rate of clearance of the antibody fusion protein from the plasma compartment.

## 1. Introduction

The non-invasive administration to brain of biologic drugs (therapeutic antibodies, neurotrophins, decoy receptors, lysosomal enzymes) is not possible, owing to the lack of transport of these large molecule pharmaceuticals through the blood–brain barrier (BBB). One approach to the BBB delivery of biologics is the re-engineering of the drug as an IgG fusion protein. The IgG domain of the fusion protein is a monoclonal antibody (MAb) that targets an exofacial epitope on certain endogenous peptide receptor-mediated transcytosis (RMT) systems expressed on the BBB membrane. The IgG domain of the fusion protein acts as a molecular Trojan horse to ferry the biologic into the brain, as binding of the IgG domain of the fusion protein to the BBB receptor triggers RMT of the IgG-biologic fusion protein through the BBB [1]. Endogenous RMT systems on the BBB that are potential conduits for biologic delivery include the insulin receptor (IR), the transferrin receptor type 1 (TfR), the leptin receptor (LEPR), and the insulin-like growth factor (IGF) receptor (IGFR), as these receptors normally serve to mediate the BBB transport of the respective endogenous ligand, insulin, transferrin (Tf), leptin, or the IGFs [1]. The first BBB Trojan horse fusion protein to enter clinical trials was a fusion protein of a MAb against the human IR (HIR), designated HIRMAb, and the lysosomal enzyme, α-L-iduronidase (IDUA), which is mutated in Type I Mucopolysaccharidosis (MPSI), and this HIRMAb-IDUA fusion protein is designated valanafusp alfa [2]. An MAb targeting the BBB TfR is designated a TfRMAb. A TfRMAb fusion protein, pabinafusp alfa, was tested in phase II and III clinical trials for MPS Type II (MPSII), and this fusion protein comprised the TfRMAb and the lysosomal enzyme mutated in MPSII, which is iduronate 2-sulfatase (IDS) [3,4,5]. Another TfRMAb-IDS fusion protein [6], DNL310, is in clinical trials for MPSII [NCT04251026], and a TfRMAb-gantenerumab fusion protein [7], RO7126209, is in clinical trials for Alzheimer’s disease [NCT04639050].

In addition to the IgG BBB Trojan horses that are currently in clinical trials, there are other TfRMAb-based brain delivery systems that are in preclinical development. TfRMAb-derived bispecific antibodies are being developed as novel radiopharmaceuticals for imaging the brain with positron emission tomography [8,9,10,11]. TfRMAb-targeted liposomes [12,13,14,15] and TfRMAb-targeted nanoparticles [16,17,18] are under development for brain drug delivery. Non-IgG delivery systems targeting the BBB TfR include cystine-dense peptides, which have a high affinity for the TfR [19]. Novel IgG-based delivery systems targeting the BBB TfR include binding sites engineered in the Fc region [6,20], variable domain of IgG (VNAR) single domain TfRMAbs [21,22], and a single chain Fv (ScFv) TfRMAb fused to an albumin-binding domain, which is fused to a therapeutic affibody [23].

The affinity of the BBB Trojan horses targeting the TfR range over several log orders of magnitude. High affinity TfRMAbs have a dissociation constant (K_D_) of binding to the TfR ranging from 0.1 to 3 nM [21,22,23,24,25,26,27,28]. Moderate-affinity TfRMAbs have a KD of binding to the TfR ranging from 14 to 76 nM [26,27,29,30,31,32]. Low-affinity TfRMAbs have a KD of binding to the TfR ranging from 111 to 300 nM [6,20,33,34]. The comparison of brain uptake of TfRMAbs with different affinity for the TfR is typically evaluated at a high injection dose (ID), e.g., 30–50 mg/kg, which produces plasma concentrations of the TfRMAb that selectively saturate the binding sites on the BBB TfR with the high-affinity TfRMAb.

In an effort to better understand the mechanisms of BBB transport of a TfRMAb, a HIRMAb, or the fusion proteins generated from these antibodies, a mathematical model has been recently developed [35]. This model was used in conjunction with experimental observations on the brain uptake of either a humanized TfRMAb [24], or a HIRMAb-IDUA fusion protein [36] in the Rhesus monkey [35]. In the present review, the results of this mathematical model are analyzed with respect to the role of the endogenous ligand, Tf or insulin, the role of high and low affinity of the TfRMAb for the BBB receptor, and the extent to which the pharmacokinetics of plasma clearance determines the brain exposure of the fusion protein.

## 2. Blood–Brain Barrier Transport of Endogenous Ligands: Transferrin and Insulin

### 2.1. Structure of the Human Transferrin Receptor-Holo Transferrin Complex

**Figure 1:**
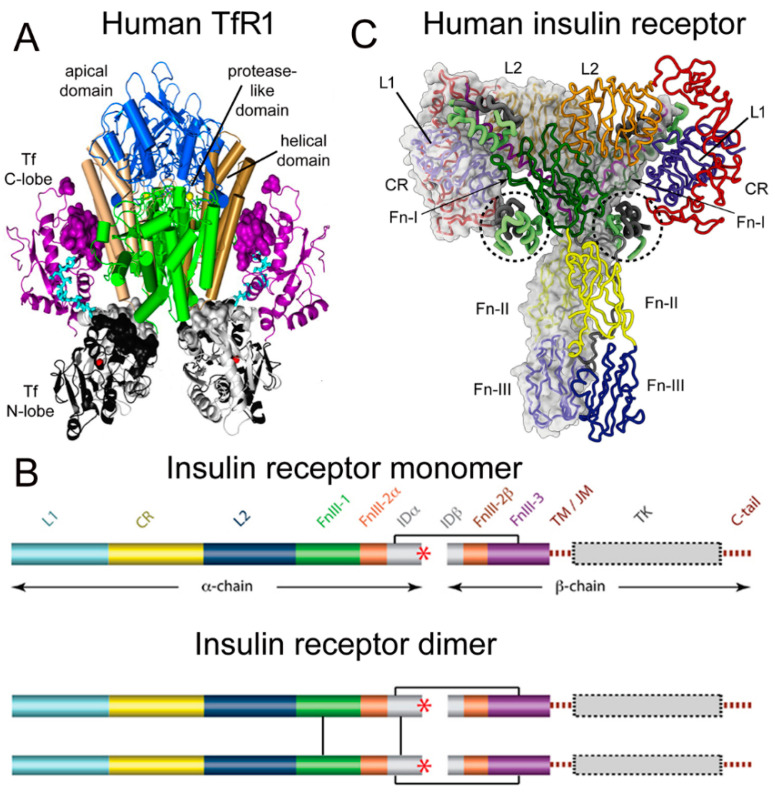
(**A**) Three-dimensional structure of the complex of the human TfR ECD and holo-Tf. The tetrameric complex is comprised of 2 TfRs and 2 holo-Tf molecules. The cell surface is at the bottom of the structure and the apical domain (blue) is at the top; the 2 protease-like domains are shown in green and the helical domain is shown in brown/tan. The N-lobe and C-lobe of Tf are shown in gray/black and purple, respectively. The Fe^+3^ bound within the N-lobe is shown in red; the linker between the N and C lobes of Tf is cyan. Reproduced with permission from [37]. (**B**) Two-dimensional structure of the human IR as a monomer (top) and a dimer (bottom). A single disulfide bond joins the alpha and beta chains of each monomer, and the dimer is formed by 2 disulfide bonds between each alpha chain. Reproduced from [42], Copyright© 2011 licensed under Creative Commons Attribution License (CC-BY). (**C**) Three-dimensional structure of the complex of the human IR and insulin. The structure is comprised of the IR dimer and 4 bound insulin molecules. Insulin bound to the second site formed by the FnIII-1/FnIII-2 domains is encircled. Reproduced with permission from [43], Copyright© 2021 Elsevier, as reported in [44]. The IR domains in panels B and C are defined in the text.

Transferrin (Tf) is a 679 amino acid bilobular protein comprised of an N-lobe (amino acids 1–331) and a C-lobe (amino acids 339–679), joined by a short linker (amino acids 332–338), and both lobes bind 1 ferrous (Fe^+3^) atom [37]. There are two transferrin receptors, TfR1 and TfR2, which are products of separate genes [38]. The TfR expressed at the BBB was identified with a BBB genomics investigation as TfR1 [39]. The crystal structure at a resolution of 3.2 angstroms was reported for the complex of the human TfR1 extracellular domain (ECD) and holo-Tf [37]. The human TfR1 ECD was expressed in baby hamster kidney (BHK) fibroblasts, and the human Tf was also expressed in BHK cells [37]. The Tf was mutated (Y426F, Y517F) to eliminate iron binding to the C-lobe, and the Tf was also mutated (N413D, N611D) to eliminate Tf N-linked glycosylation [37]. The hetero-tetrameric Tf-TfR complex is formed by two receptors and two holo-Tf molecules [37]. The TfR1 is a 760 amino acid protein comprised of multiple domains, including the intracellular amino terminal domain (amino acids 1–67), the transmembrane domain (amino acids 68–88), a stalk domain, which forms disulfide bonds between two receptors (amino acids 89–120), two protease-like domains (amino acids 121–188 and 384–606), an apical domain (amino acids 189–383), and a helical domain (amino acids 607–760) [37]. Amino acids 121–760 form the monomeric ECD of the TfR1. Transferrin in plasma exists in three forms: about 40% is apo-Tf, which does not bind to the TfR1 at physiologic pH; about 30% is diferric holo-Tf; and about 30% is mono-ferric Tf [37]. The affinity of diferric Tf for the TfR1 is ~6-fold greater than the affinity of monoferric Tf [40]. The concentration of Tf in human plasma is 45,000 nM [41], and the concentration of holo-Tf is about 25,000 nM. The plasma concentration of holo-Tf is nearly 1000-fold greater than the TfR1 concentration at the brain capillary endothelium in vivo, which is 40 nM [35]. The optimal TfRMAb binding site on the TfR is the apical domain, as holo-Tf binds to the protease-like and helical domains of the TfR1 as shown in Figure 1A.

### 2.2. Structure of the Human Insulin Receptor-Insulin Complex

There are two human insulin receptors, designated IR-A (short form) and IR-B (long form), which are derived from a single gene by alternate processing of the primary transcript. In IR-A, which is primarily expressed in cancer and fetal tissues [45], exon 11 is deleted, resulting in a 12 amino acid truncation at the carboxyl terminus of the alpha chain, which corresponds to the α-CT domain of IR-B. IR-B is the isoform predominantly expressed in tissues [45]. Following removal of a 27 amino acid signal peptide, IR-B is encoded as a 1355 amino acid polypeptide, which is proteolytically cleaved to the alpha chain, amino acids 1–731 (not counting the signal peptide), and the beta chain, amino acids 736–1355 [46]. This separation into alpha and beta chains occurs at a furin cleavage site, RKRR [47], which corresponds to amino acids 732–735, and this sequence is removed in the cleavage. The cleavage into the separate alpha and beta chains is shown in Figure 1B (top). The alpha chain is formed by the first leucine-rich (L1) domain, the cysteine-rich (CR) domain, the second leucine-rich (L2) domain, the first fibronection III domain (FnIII-1), the first part of the second fibronection III domain (FnIII-2α), and the first part of the insert domain (IDα); the final 12 amino acids of the alpha chain is the αCT domain, which is involved in insulin binding [46]. The beta chain is formed by the second part of the insert domain (IDβ), the second part of the FnIII-2 domain (FnIII-2β), the third fibronectin domain (FnIII-3), the transmembrane (TM) domain, the juxtamembrane (JM) domain, the tyrosine kinase (TK) domain, and the carboxyl terminus (Figure 1B, top). An inter-chain disulfide bond joins the alpha and beta chains, and two additional disulfides between the two alpha chains form the hetero-tetrameric structure of the IR (Figure 1B, bottom). The ECD of the IR, which is approximately 900 amino acids in length, is formed by cleavage near the TM domain and includes all of the alpha chain and the amino terminal portion of the beta chain. The crystal structure of the ECD of the human IR complexed with monoclonal antibodies was originally produced [48]. Recently, the three-dimensional structure of the complex of insulin and the IR tetrameric structure was generated with cryo electron microscopy [44,49], as recently reviewed [43], and this structure is shown in Figure 1C. The structure of the insulin/IR complex reveals each IR monomer binds two insulin molecules, so that the IR dimer shown in Figure 1C binds four insulin molecules; two insulins are bound to the classical high-affinity binding site formed by interaction of the L1 and αCT domains of each alpha subunit and two insulins are bound to a low-affinity second site formed by interactions of the FnIII-1 and FnIII-2 domains of each alpha subunit (Figure 1C). Insulin is synthesized as a proinsulin precursor in pancreatic beta cells, and proinsulin is cleaved to 2 insulin subunits, the 21 amino acid A-chain and the 30 amino acid B-chain, which are joined together by 2 disulfide bonds [43]. The fasting plasma insulin concentration is about 0.3 nM in humans and primates [50,51]. The plasma concentration of insulin is ~100-fold lower than the IR concentration at the brain capillary endothelium in vivo, which is 24 nM [35].

### 2.3. BBB Transport of Holo-Transferrin

**Figure 2:**
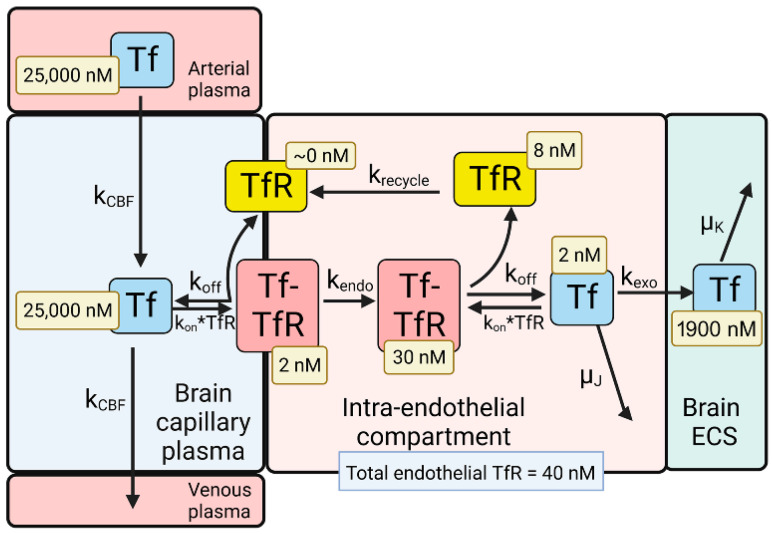
Model of transport of holo-transferrin (Tf) from the blood to the brain extracellular space (ECS) through the brain capillary endothelium, which forms the BBB in vivo. Holo-Tf in plasma binds the transferrin receptor (TfR) on the luminal endothelial membrane to form the luminal Tf-TfR complex, which is followed by endocytosis into the intra-endothelial compartment. Following dissociation of the Tf within the endothelium, the Tf undergoes exocytosis into the brain extracellular space (ECS). The model allows for estimations of the concentrations of Tf, or the TfR, in each pool in the transcytosis pathway, and these concentrations are shown in the light-yellow boxes. Adapted from [35], Copyright© 2021 licensed under Creative Commons Attribution License (CC-BY). Image created with Biorender.com.

The model solutions by numerical analysis of a partly flow-partly compartmental model of BBB holo-Tf transport have been described previously [35], and the holo-Tf model is shown in Figure 2.

The dissociation (k_off_) and association (k_on_) rate constants of holo-Tf binding to the human TfR are 0.06 min^−1^ and 0.1 nM^−1^min^−1^, respectively, which corresponds to a K_D_ = 0.6 nM [52]. The rate constants of endocytosis (k_endo_), exocytosis (k_exo_), receptor recycling (k_recycle_), and cerebral blood flow (k_CBF_) are 0.07–0.14 min^−1^ (T_1/2_ = 5–10 min), 0.14 min^−1^ (T_1/2_ = 5 min), 0.035 min^−1^ (T_1/2_ = 20 min), and 42 min^−1^ [35]. The initial conditions of the model set [Tf] = 25,000 nM and [Tf] = 0 for the concentration of Tf in the plasma and brain ECS, respectively. The experimentally observed concentration of Tf in the brain is 114 ug/gram [53], which is equal to 2000 nM, as the brain water volume is 0.7 mL/g [54]. Given a rate constant of Tf degradation in the brain of μ_K_ = 0.00014 min^−1^ (T_1/2_ = 82 h or 3.4 days), model analysis showed the Tf in the brain ECS reached an equilibrium concentration of 1900 nM. This T_1/2_ of Tf removal from the brain of 3.4 days corresponds to the plasma T_1/2_ of Tf, which is 2.5 days [55]. At steady state, the concentration of free TfR on the luminal membrane was ~0 (Figure 2), owing to the vastly greater concentration of holo-Tf in plasma, 25,000 nM, as compared to the total concentration of TfR, 40 nM, at the brain capillary endothelium [35]. Most of the endothelial TfR, 30 nM or 75% of total endothelial TfR, was localized to the intra-endothelial compartment as a Tf-TfR complex; the concentration of free Tf and free TfR within the endothelial compartment was estimated to be 2 and 8 nM, respectively (Figure 2). The concentration of the Tf-TfR complex at the endothelial luminal membrane is 2 nM, which is only 5% of the total endothelial TfR (Figure 2). The absence of free TfR at the endothelial luminal membrane indicates a TfRMAb in the plasma binds the tetrameric complex of holo-Tf and the TfR (Figure 1A), which is embedded in the endothelial plasma membrane.

### 2.4. BBB Transport of Insulin

**Figure 3:**
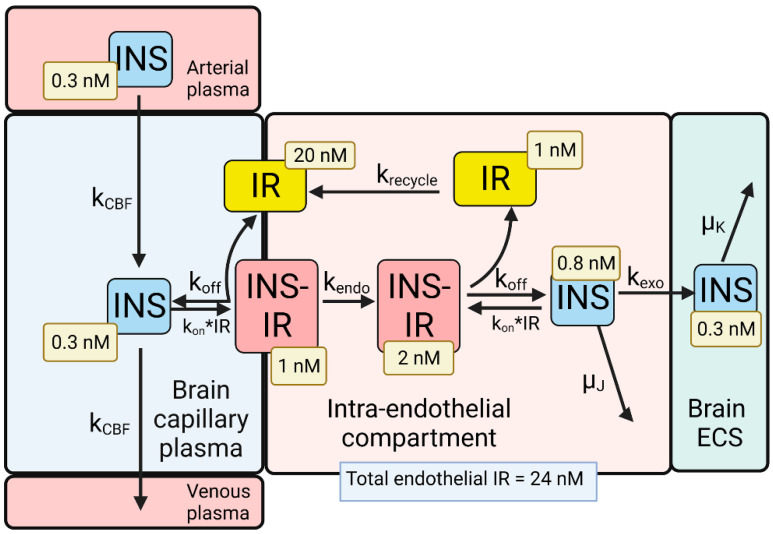
Model of transport of insulin (INS) from the blood to the brain extracellular space (ECS) through the brain capillary endothelium, which forms the BBB in vivo. INS in plasma binds the insulin receptor (IR) on the luminal endothelial membrane to form the luminal INS-IR complex, which is followed by endocytosis into the intra-endothelial compartment. Following dissociation of the INS within the endothelium, the INS undergoes exocytosis into the brain ECS. The model allowed for estimations of the concentrations of INS, or the IR, in each pool in the transcytosis pathway, and these concentrations are shown in the light-yellow boxes. Adapted from [35], Copyright© 2021 licensed under Creative Commons Attribution License (CC-BY). Image created with Biorender.com.

A partly flow-partly compartmental model of BBB transport of insulin is outlined in Figure 3.

The differential equations and insulin (INS) model solutions by numerical analysis have been described previously [35]. The dissociation (k_off_) and association (k_on_) rate constants of INS binding to the human IR are 0.26 min^−1^ and 0.1 nM^−1^min^−1^, respectively, which corresponds to a K_D_ = 2.6 nM [56]. The rate constants of endocytosis (k_endo_), exocytosis (k_exo_), and receptor recycling (k_recycle_) are 0.023 min^−1^ (T_1/2_ = 30 min), 0.035 min^−1^ (T_1/2_ = 20 min), and 0.035 min^−1^ (T_1/2_ = 20 min), respectively, as described previously [35]. The rate constant of cerebral blood flow (k_CBF_), 42 min^−1^ (T_1/2_ = 1 s), was derived from the Vp/CBF ratio, where the brain plasma volume (Vp) is 0.01 mL/g [57], and the rate of cerebral blood flow (CBF) is 0.6 mL/min/g [58]. The rate constant of INS degradation within the endothelium, μ_J_, was fixed at 0.0058 min^−1^ (T_1/2_ = 2 h), as prior work showed no insulin degradation by isolated human brain microvessels within 60 min at 37 °C [39]. The initial conditions of the model set [INS] = 0.3 nM and [INS] = 0 for the concentration of INS in the plasma and brain ECS, respectively. If the model was run from 0 to 6 h, and the rate constant of INS degradation in brain was set at μ_K_ = 0.138 min^−1^ (T_1/2_ = 5 min), then the INS in brain ECS reached an equilibrium concentration of 0.3 nM (Figure 3), which corresponds to the experimentally observed insulin concentration in the brain. The brain insulin concentration is 9.6 ± 3.4 μU/g [59], which is equivalent to 48 μU/mL, given an ECS volume in the brain of 0.2 mL/g [60]. Converting μU of insulin to fmol of insulin, based on 1 μU = 6 fmol [61], the experimentally observed brain insulin concentration is 0.3 nM. A T_1/2_ of 5 min of INS removal from brain ECS corresponds with the T_1/2_ of INS removal from plasma, which is 4–6 min [62]. At steady state, the concentration of free IR on the luminal membrane was 20 nM (Figure 3), which is 83% of the total IR, 24 nM, in the brain capillary endothelium [35]. The high concentration of free IR at the luminal membrane (Figure 3), compared to the very low level of free TfR at the luminal membrane (Figure 2), is due to the nearly 5 log orders of magnitude difference in the plasma concentration of INS, 0.3 nM, and holo-Tf, 25,000 nM. The concentration of free INS and free IR within the endothelial compartment was estimated to be 0.8 and 1 nM, respectively (Figure 3). These modeling studies for INS indicate an IRMAb in the plasma primarily binds to the unbound IR, rather than the INS-IR complex.

## 3. Blood–Brain Barrier Transport of a Transferrin Receptor or Insulin Receptor Antibody

### 3.1. Receptor Binding Sites of TfR and IR Antibodies

The TfRMAb modeled in these studies is a humanized version [24] of the murine 128.1 MAb against the human TfR, which was originally isolated from a myeloma line [63]. The murine form of the 128.1 antibody cross reacts with the TfR in the African green monkey, an Old World primate, and undergoes transport through the BBB of this primate in vivo [64]. The 128.1 TfRMAb does not compete with holo-Tf for binding to the TfR1 [65], and the epitope of this antibody lies in the apical domain of the TfR1 between Ser-324 and Ser-368 [66]. The amino acid sequence of this epitope is 95% conserved in the TfR1 of humans (NP_001121620), Rhesus monkeys (NP_001244232), the cynomolgus monkeys (XP_005545315), and the African green monkey (AFD18259); the 2 amino acid mismatches lie at the far amino terminal and far carboxyl terminal sequences of this 45 amino acid epitope. The kinetics of binding of the chimeric form of the 128.1 TfRMAb to the human TfR1 ECD was determined with surface plasmon resonance (SPR), which showed the k_on_ = 3 × 10^5^ M^−1^sec^−1^ [66]. Since k_on_ rates are up to 7-fold higher at 37 °C [67,68], the in vivo k_on_ of the 128.1 antibody binding to the TfR1 approximates 10^6^ M^−1^sec^−1^.

The HIRMAb modeled in these studies is a HIRMAb-IDUA fusion protein (valanafusp alfa), where the IDUA enzyme was fused to the carboxyl terminus of each heavy chain of a chimeric HIRMAb [2]. This human/mouse chimeric HIRMAb was genetically engineered [69] after determining the sequences of the heavy and light chain variable regions of the murine 83–14 MAb against the HIR, which was originally generated from a myeloma line [70]. The epitope of the murine 83–14 antibody lies between amino acids 469 and 592 of the alpha chain of the HIR [70], which corresponds to the first fibronectin domain, FnIII-1 (Figure 1). This HIRMAb cross reacts with the Rhesus monkey IR [71], and the sequence of the 83–14 epitope is 100% conserved in the IR of humans (P06213), Rhesus monkeys (AFE71352), and cynomolgus monkeys (XP_005587797). The brain uptake in the Rhesus monkey is 2.0 ± 0.1%/100 g brain for the chimeric HIRMAb [69], and is 1.2 ± 0.2%/100 g brain for the HIRMAb-IDUA fusion protein [36]. Brain uptake in the primate is expressed per 100 g brain, because the weight of the primate brain is 100 g [71]. The binding site of the 83–14 antibody on the HIR is spatially removed from the primary insulin binding site at the interface of the L1 and αCT domains (Figure 1C). However, the 83–14 antibody has both allosteric agonist and antagonist effects, and both inhibits insulin binding and stimulates glucose uptake by cells in vitro [72]. Whereas insulin binding to the alpha subunit of the HIR triggers auto-phosphorylation of the beta subunit, the 83–14 antibody induces auto-phosphorylation only at high antibody concentrations [72]. In humans, IV infusion of 1–3 mg/kg of the HIRMAb-IDUA fusion protein in 5% dextrose causes mild reversible hypoglycemia in only 2.1% of >500 infusions administered over the course of 1 year of treatment [2]. Chronic administration of the HIRMAb-IDUA fusion protein has no effect on glycemic control [2]. The murine 83–7 antibody binds the HIR [73], and the binding site of this antibody is within the CR domain between amino acids 233 and 281 [48]. The 83–7 antibody does not inhibit insulin binding to the HIR [72]. However, the HIR-mediated uptake of the 83–14 antibody by capillaries isolated from human autopsy brain is >10-fold higher than the brain capillary uptake of the 83–7 antibody [71]. Binding of the 83–7 antibody to the HIR expressed in isolated human brain capillaries has allosteric effects on the HIR, as the presence of the 83–7 antibody inhibits binding of the 83–14 antibody to the HIR at the human BBB [71].

### 3.2. Kinetics of BBB Transport of a TfRMAb in the Rhesus Monkey

**Figure 4:**
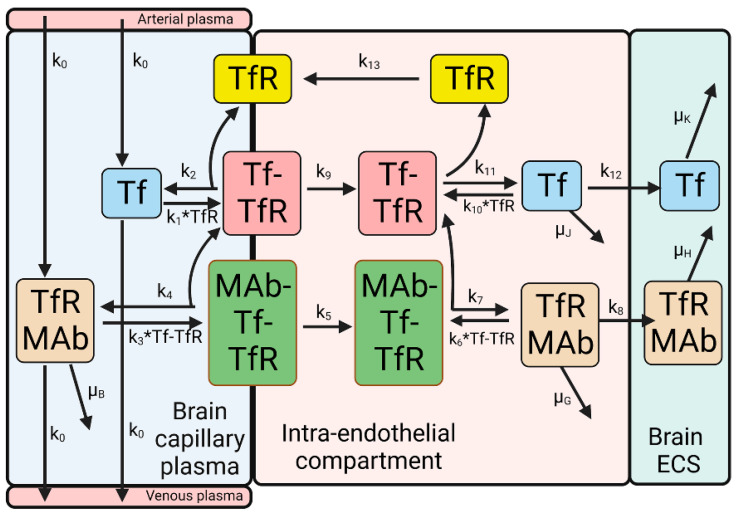
Model of simultaneous transport of holo-Tf and a TfRMAb from the blood to the brain ECS across the capillary endothelium. The model includes rate constants of Tf and TfRMAb dissociation and association with the TfR or Tf-TfR complex; rate constants for Tf-TfR (k_9_) and MAb-Tf-TfR (k_5_) endocytosis into the intra-endothelial compartment; rate constants for Tf (k_12_) and TfRMAb (k_8_) exocytosis into the brain ECS; the rate constant (k_13_) for TfR recycling back to the luminal endothelial membrane, and the rate constant (k_0_) for cerebral plasma flow. The model includes a rate constant for TfRMAb removal from plasma (μ_B_) via mechanisms other than BBB transport, e.g., binding to erythrocytes; a rate constant for Tf (μ_J_) or TfRMAb (μ_G_) removal from the brain endothelial compartment via a mechanism other than exocytosis, and a rate constant for Tf (μ_K_) or TfRMAb (μ_H_) removal from brain ECS, e.g., due to either degradation or efflux back to blood. Adapted from [35], Copyright© 2021 licensed under Creative Commons Attribution License (CC-BY). Image created with Biorender.com.

A partly flow-partly compartmental model of the simultaneous BBB transport of holo-Tf and a TfRMAb has been described previously [35], and is outlined in Figure 4.

The TfRMAb/Tf model shown in Figure 4 is comprised of 12 differential equations, 23 input parameters, and 11 output variables; the model was solved by numerical analysis using the NDSolve program of Wolfram Mathematica [35]. The model assumes that the TfRMAb dissociates from the Tf-TfR complex prior to exocytosis [35]. This is consistent with the model of albumin RMT through the lung endothelium, where the albumin ligand, and the albumin receptor, gp60 (albondin), are endocytosed as a complex, followed by localization of free albumin in a pre-exocytosis vesicle [74]. A comparison has been made of the exocytosis at synapses for neurotransmitter release, and exocytosis at the brain endothelium for transcytosis [75]. Exocytosis from the brain capillary endothelial cell involves fusion of the ligand-bearing vesicle with the abluminal endothelial membrane, and this fusion is mediated by multiple intracellular proteins [75].

Estimates of rate constants (k_1_, k_2_, k_10_, k_11_) of Tf dissociation or association with the TfR are available from the literature [52]. However, information on the rate constants of endocytosis (k_5_, k_9_), exocytosis (k_8_, k_12_), and receptor recycling (k_13_) at the BBB in vivo are not available from the literature and were estimated by the model shown in Figure 4 [35]. In addition, the rate constant of TfRMAb association (k_3_, k_6_) with the Tf-TfR complex at the BBB in vivo is uncertain. First, this rate constant is typically measured in vitro by SPR using the soluble TfR1 ECD monomer unbound by Tf. However, at the brain endothelial luminal membrane in vivo, the TfRMAb binds to a hetero-tetrameric complex of two Tf molecules bound to a TfR disulfide-linked receptor dimer (Figure 1A), which is embedded in the plasma or endosomal membranes. Second, the TfR1 surface density at the brain capillary in vivo, 0.03 fmol/mm^2^ [35], is >100-fold lower than the receptor surface density used in SPR experiments [76,77]. Unknown rate constants were estimated by fitting the mathematical model to experimentally observed brain uptake of the TfRMAb [35]. The experimentally determined uptake has been reported previously for the humanized 128.1 TfRMAb in the Rhesus monkey at 2 h after IV administration [24]. The rate constants of MAb-Tf-TfR endocytosis (k_5_), TfRMAb exocytosis (k_8_), TfR recycling back to the luminal membrane (k_13_), and TfRMAb association (k_3_) and dissociation (k_7_) with the Tf-TfR complex at the luminal membrane were varied until the predicted brain uptake of the TfRMAb, measured as %ID/100 g brain at 2 h after IV administration, matched the model-predicted brain uptake. It is assumed the rate constants of TfRMAb association (k_6_) and dissociation (k_7_) with the intracellular Tf-TfR complex are identical to comparable rate constants (k_3_, k_4_) for the Tf-TfR complex at the luminal membrane. The k_4_/k_3_ ratio, or the k_7_/k_6_ ratio, which is the dissociation constant (K_D_), was fixed at the value of 0.36 nM [35].

The injection dose (ID), 0.2 mg/kg, used in the modeling studies was identical to the ID used in the in vivo brain uptake studies in the Rhesus monkey [24]. The initial conditions of the model were determined with the Tf transport model (Figure 2), and include (i) luminal free TfR = 0; (ii) luminal Tf-TfR complex = 2 nM; (iii) intracellular free TfR = 8 nM; (iv) intracellular Tf-TfR complex = 30 nM; and (v) plasma holo-Tf = 25,000 nM. The total endothelial TfR concentration, 40 nM, was estimated from literature values of the expression of the TfR1 at the human brain microvessel, and the portion of brain that is comprised of brain capillary protein, as described previously [35]. The TfRMAb concentration in the brain capillary plasma, designated B(t), is defined by the equation, $B\left(t\right)=A_{0}e^{-aT}$, where $A_{0}$ = maximal plasma concentration (Cmax) of the TfRMAb, α = the rate constant of monoexponential decay in plasma, and T = time after IV administration for a given injection dose (ID) of the TfRMAb [35].

**Figure 5:**
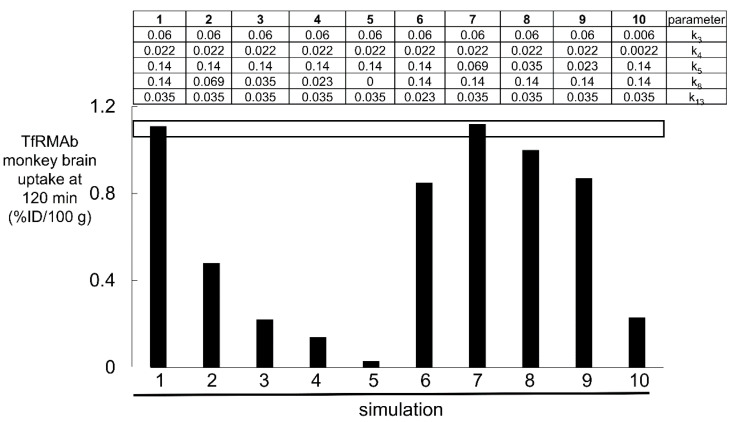
Simulations 1–10, wherein certain parameters (k_3_, k_4_, k_5_, k_8_, k_13_) shown in Figure 4 were varied in each simulation. The units of k_4_, k_5_, k_8_, and k_13_ are min^−1^, and the units of k_3_ are nM^−1^min^−1^ [35]. The value of each of the 5 parameters for each of the 10 simulations are shown in the top part of the figure. The open horizontal bracket is the experimentally observed brain uptake of the TfRMAb in the Rhesus monkey, 1.2 ± 0.1%ID/100 g brain at 2 h after IV administration of an ID of 0.2 mg/kg [24]. The parameters of only simulations 1 and 7 fit the experimentally observed brain uptake. Adapted from [35], Copyright© 2021 licensed under Creative Commons Attribution License (CC-BY).

The results of 10 simulations of the TfRMAb mathematical model (Figure 4), and the respective parameters used in each of the 10 simulations with the TfRMAb model, are shown in Figure 5.

Over the course of these 10 simulations of the TfRMAb model, the rate constants for endocytosis (k_5_), exocytosis (k_8_), and receptor recycling (k_13_), and TfRMAb association (k_3_, k_6_) with the Tf-TfR complex were varied. Simulations 1 and 7 show that the model fits the experimentally observed brain uptake when the endocytosis rate constant (k_5_) is 0.069–0.14 min^−1^ (T_1/2_ = 5–10 min), the exocytosis rate constant (k_8_) is 0.14 min^−1^ (T_1/2_ = 5 min), the TfR recycling rate constant (k_13_) is 0.035 min^−1^ (T_1/2_ = 20 min), the rate constant of TfRMAb association with the membrane-bound Tf-TfR complex (k_3_) is 0.06 nM^−1^min^−1^, which is equivalent to 10^6^ M^−1^sec^−1^, and the rate constant of TfRMAb dissociation from the membrane-bound Tf-TfR complex (k_4_) is 0.022 min^−1^ (T_1/2_ = 31 min) [35]. The rate constant of the TfRMAb dissociation (k_4_) from the membrane-bound Tf-TfR complex was computed from k_4_ = k_3_·K_D_, where K_D_ = 0.36 nM, which is the dissociation constant of TfRMAb binding to the human TfR1 [24]. Simulations 2, 3, and 4 show that when the exocytosis rate constant (k_8_) is lowered to 0.069 min^−1^ (T_1/2_ = 10 min), 0.035 min^−1^ (T_1/2_ = 20 min), and 0.023 (T_1/2_ = 30 min) min^−1^, respectively, the predicted brain uptake is low compared to the observed brain uptake of the TfRMAb. Simulation 5 shows that when the antibody undergoes no exocytosis into brain ECS, as represented by k_8_ = 0, the predicted brain uptake is near zero, which is >10-fold lower than the experimentally observed brain uptake (Figure 5). Simulation 6 shows that when the recycling rate constant (k_13_) is reduced to 0.023 min^−1^ (T_1/2_ = 30 min), brain uptake is reduced compared to the observed brain uptake. Simulations 8 and 9 show that when the rate constant of endocytosis (k_5_) is reduced to 0.035 min^−1^ (T_1/2_ = 20 min) and 0.023 (T_1/2_ = 30 min) min^−1^, respectively, the predicted brain uptake is reduced compared to the observed brain uptake. Simulation 10 shows that if the association and dissociation rate constants (k_3_, k_4_) of TfRMAb binding to the Tf-TfR complex are each reduced 10-fold, the predicted brain uptake is much lower than the observed brain uptake. 

The value for μ_B_ was set at 0.00096 min^−1^ (T_1/2_ = 12 h), as there is no expression of the TfR1 on mature erythrocytes [78]. The value for μ_G_, the rate constant of TfRMAb degradation within the intra-endothelial compartment, was set at 0.0058 min^−1^ (T_1/2_ = 2 h). Previous simulations showed increasing the value of μ_G_ had no effect on the brain TfRMAb concentration until μ_G_ was increased to 0.138 min^−1^ (T_1/2_ = 5 min), where the rate of TfRMAb degradation in the endothelium approximates the rate of TfRMAb exocytosis into brain ECS [35]. The value for μ_H_, the rate constant of TfRMAb removal from brain ECS, via either degradation or efflux back to blood, was set at 0.00096 min^−1^ (T_1/2_ = 12 h), which approximates the T_1/2_ of turnover of TfRMAbs in the brain reported in the literature [79,80].

### 3.3. Kinetics of BBB Transport of a HIRMAb-IDUA Fusion Protein in the Rhesus Monkey

**Figure 6:**
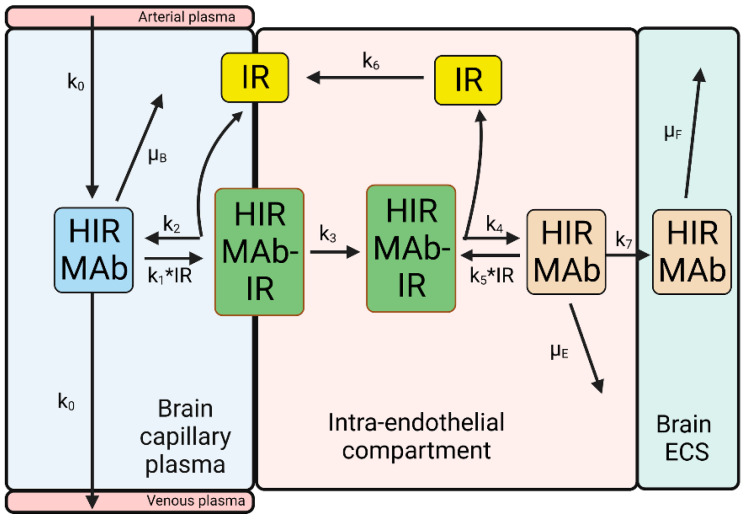
Model of transport of HIRMAb-IDUA fusion protein from the blood to the brain ECS across the capillary endothelium. The model includes rate constants of HIRMAb-IDUA dissociation from (k_2_, k_4_) and association with (k_1_, k_5_) the IR; rate constant for endocytosis (k_3_) of the complex of the IR and HIRMAb-IDUA into the intra-endothelial compartment; rate constant for HIRMAb-IDUA exocytosis (k_7_) into the brain ECS; the rate constant (k_6_) for IR recycling back to the luminal endothelial membrane; and the rate constant (k_0_) for cerebral plasma flow. The model includes rate constants for HIRMAb-IDUA removal from plasma (μ_B_) via erythrocyte binding, removal from the intra-endothelial compartment (μ_E_) via a mechanism other than exocytosis, and removal from the brain ECS (μ_F_) via either degradation or efflux back to the blood. Adapted from [35], Copyright© 2021 licensed under Creative Commons Attribution License (CC-BY). Image created with Biorender.com.

A model of the BBB transport of the HIRMAb-IDUA fusion protein is shown in Figure 6.

The IRMAb model in Figure 6 has fewer parameters than the TfRMAb model in Figure 4. The reduced complexity of the IRMAb model is due to the lack of simultaneous transport of the IRMAb and the endogenous receptor ligand, insulin. Since the concentration of plasma insulin, 0.3 nM, is 100-fold lower than the total IR concentration at the brain endothelium, 24 nM [35], the concentration of the INS-IR complex at the luminal endothelial membrane is <5% of the total endothelial IR (Figure 3). Consequently, the HIRMAb binds the free IR at the luminal endothelial membrane (Figure 6). The IRMAb model was tested with the HIRMAb-IDUA fusion protein. The brain uptake of this fusion protein has been measured in the Rhesus monkey at 2 h following the IV administration of an ID of 0.1 mg/kg [36].

**Figure 7:**
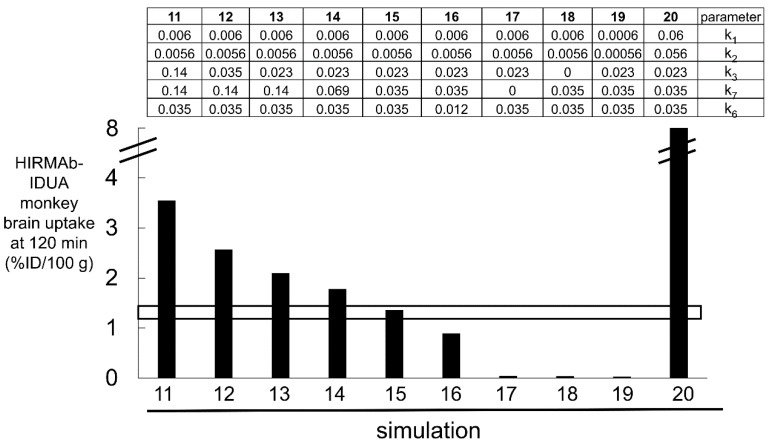
Simulations 11–20, wherein certain parameters (k_1_, k_2_, k_3_, k_6_, k_7_) shown in Figure 6 were varied. The units of k_2_, k_3_, k_6_, and k_7_ are min^−1^, and the units of k_1_ are nM^−1^min^−1^ [35]. The value of each of the 5 parameters for each of the 10 simulations are shown in the top part of the figure. The open horizontal bracket is the experimentally observed brain uptake of the HIRMAb-IDUA fusion protein in the Rhesus monkey, 1.2 ± 0.2%ID/100 g brain at 2 h after IV administration of an ID of 0.1 mg/kg [36]. The parameters of only simulation 15 fit the experimentally observed brain uptake. Adapted from [35], Copyright© 2021 licensed under Creative Commons Attribution License (CC-BY).

Estimates of the rate constants of an HIRMAb association, 1.0 × 10^5^ M^−1^sec^−1^, which is equivalent to 0.006 nM^−1^min^−1^, and dissociation, 0.013 min^−1^, with the soluble HIR ECD are available from the literature using SPR [81]. However, it is uncertain if such estimates are representative of HIRMAb binding to the IR that is embedded in the endothelial luminal membrane in vivo. In addition, there is no information on the rate constant of IR endocytosis (k_3_) at the BBB, HIRMAb exocytosis into the brain ECS (k_7_), or IR receptor recycling within the intra-endothelial compartment (k_6_), and these parameters were estimated by fitting the IRMAb model (Figure 6) to the experimentally observed brain uptake of the HIRMAb-IDUA fusion protein in the Rhesus monkey [36]. The results of 10 simulations, numbered 11–20, of the IRMAb model are shown Figure 7, as reported previously [35].

Over the course of the 10 simulations described in Figure 7, the rate constants for endocytosis (k_3_), exocytosis (k_7_), and receptor recycling (k_6_), and HIRMAb-IDUA association (k_1_, k_5_) with the IR were varied. Only the parameters of simulation 15 produced a match between the experimentally observed brain uptake and the brain uptake predicted by the model (Figure 7). For the starting parameters, represented by simulation 11, the association rate constant (k_1_) was set at 0.006 nM^−1^min^−1^ [81], and the dissociation rate constant (k_2_), 0.0056 min^−1^, was computed from k_2_ = k_1_·K_D_, where K_D_ is the dissociation constant, 0.93 nM, of HIRMAb-IDUA fusion protein binding to the HIR ECD [35]. The endocytosis (k_3_), exocytosis (k_7_), and receptor recycling (k_6_) rate constants in simulation 11 were taken from simulation 1 of the TfRMAb model (Figure 5). These parameters for simulation 11 predicted a brain uptake of the HIRMAb-IDUA fusion protein that was 3-fold higher than the experimentally observed brain uptake (Figure 7). In simulations 12 and 13, the endocytosis rate constant (k_3_) was reduced to 0.035 min^−1^ (T_1/2_ = 20 min) and 0.023 min^−1^ (T_1/2_ = 30 min), respectively, and these parameters decreased the predicted brain uptake, which was still higher than the observed uptake (Figure 7). In simulations 14 and 15, the endocytosis rate constant (k_3_) was fixed at 0.023 min^−1^ (T_1/2_ = 30 min), and the exocytosis rate constant (k_7_) was reduced to 0.069 min^−1^ (T_1/2_ = 10 min) and 0.035 min^−1^ (T_1/2_ = 20 min), respectively. The parameters of simulation 15 matched the predicted brain uptake with the experimentally observed brain uptake of the HIRMAb-IDUA fusion protein (Figure 7). In simulation 16, the rate constants of endocytosis and exocytosis from simulation 15 were used, but the rate constant (k_6_) of receptor recycling was reduced to 0.012 min^−1^ (T_1/2_ = 60 min), and this resulted in a reduced brain uptake (Figure 7). In simulation 17, the rate constant of exocytosis (k_7_) = 0, which assumes the HIRMAb-IDUA fusion protein is only trapped inside the endothelium without any delivery into the brain ECS. In simulation 18, the rate constant of endocytosis (k_3_) = 0, which assumes the HIRMAb-IDUA fusion protein is only bound at the luminal membrane of the capillary endothelium without any subsequent endocytosis into the endothelial cell. In either the ‘no exocytosis’ or ‘no endocytosis’ simulations, the predicted brain uptake of the fusion protein is nearly zero, and >90% lower than the experimentally observed uptake (Figure 7). In simulation 19, the endocytosis, exocytosis, and recycling parameters of simulation 15 are used, but the rate constants of association with (k_1_) and dissociation from (k_2_) the IR are each reduced 10-fold relative to simulation 15, and this simulation predicts a level of brain uptake >90% reduced from the experimentally observed uptake (Figure 7). In simulation 20, the endocytosis, exocytosis, and recycling parameters of simulation 15 are used, but the rate constants of association with (k_1_) and dissociation from (k_2_) the IR are each increased 10-fold relative to simulation 15, and this simulation predicts a level of brain uptake nearly 8-fold higher than the experimentally observed uptake (Figure 7). The large difference in predicted brain uptake between simulations 19 and 20, which is caused by a 100-fold increase in the rate constants of association or dissociation of the fusion protein with the IR, with a fixed K_D_ of 0.93 nM, is due to the very low plasma concentration of the fusion protein that is associated with the low ID, 0.1 mg/kg, used in these simulations. This low ID was used in the simulations because this was the ID used in the experimental studies on the fusion protein uptake by the brain in the Rhesus monkey [36].

Simulation 15 shows that the IRMAb model fits the experimentally observed brain uptake when the endocytosis rate constant (k_3_) is 0.023 min^−1^ (T_1/2_ = 30 min), the exocytosis rate constant (k_7_) is 0.035 min^−1^ (T_1/2_ = 20 min), the IR recycling rate constant (k_6_) is 0.035 min^−1^ (T_1/2_ = 20 min), the rate constant of IRMAb association with the membrane-bound IR (k_1_) is 0.006 nM^−1^min^−1^, which is equivalent to 10^5^ M^−1^sec^−1^, and the rate constant of IRMAb dissociation from the membrane-bound IR (k_2_) is 0.0056 min^−1^ (T_1/2_ = 120 min) [35]. It is assumed the rate constants of IRMAb association with (k_5_), and dissociation from (k_4_), the intracellular IR are identical to k_1_ and k_2_, respectively [35].

### 3.4. Summary of the Kinetics of RMT via the Transferrin Receptor and Insulin Receptor

The results of fitting the TfRMAb mathematical model, outlined in Figure 4, and the IRMAb model, outlined in Figure 6, to the experimentally observed brain uptake of a TfRMAb or HIRMAb-IDUA fusion protein are shown in Figure 5 and Figure 7, respectively, as reported previously [35]. These modeling studies provide estimates of the rates of endocytosis, exocytosis, and receptor recycling at the brain capillary endothelium in vivo for transport via either the TfR or the IR. An alternative methodology for the estimation of these parameters of endocytosis, exocytosis, and receptor recycling within the brain capillary endothelium is with an in vitro BBB model in cell culture. However, in vitro BBB models are not representative of the BBB in vivo, owing to marked downregulation of tissue-specific gene expression at the brain capillary endothelium in cell culture [39]. The best fit results for BBB transport of the TfRMAb, and the HIRMAb-IDUA fusion protein, have been reported previously [35]. The parameter estimates for TfR and IR endocytosis, exocytosis, and receptor recycling at the brain capillary endothelium align with comparable studies reported in the literature. The kinetics of endocytosis, exocytosis, or receptor recycling at the brain capillary endothelium for the IR or TfR are summarized in Table 1.

With respect to the TfR, the T_1/2_ of TfR endocytosis in cells ranges from 4–6 min [82,83,84], and the T_1/2_ of TfR recycling is 17 min [82]. The T_1/2_ of exocytosis of 5 min is consistent with in vivo measurements of the rate of BBB transcytosis of either [^125^I]-holo-Tf or a [^3^H]-TfRMAb. Both holo-Tf and the high-affinity TfRMAb rapidly penetrate into the post-vascular volume of the rat brain following a 10 min internal carotid artery infusion [85], and these results were confirmed by emulsion autoradiography of rat brain removed after only 5 min of internal carotid artery infusion [85]. With respect to the IR, the T_1/2_ of IR endocytosis in rat liver cells in vivo is 30 min for the unoccupied IR [86]. The T_1/2_ of endocytosis of the HIRMAb by isolated brain microvessels is 15–30 min [71]. The T_1/2_ of IR recycling within the endothelium of 20 min compares to the T_1/2_ of receptor recycling in rat liver in vivo [35]. The T_1/2_ of exocytosis via the IR of 20 min corresponds with prior in vivo carotid artery infusion of [^125^I]-insulin in the rabbit; emulsion autoradiography of the brain after a 10 min arterial infusion demonstrated insulin movement well into brain parenchyma [87]. HPLC of acid ethanol extracts of brain showed the radioactivity in the brain was unmetabolized insulin [87]. The rapidity of the RMT process via either the TfR or IR may be related to the short distance that is traversed by the RMT pathway at the brain capillary endothelium. The thickness of the endothelial cell in the brain, 0.3 microns [88], is only 3% of the thickness, 10 microns [89], of the choroid plexus epithelium. The intracellular volume of the brain capillary endothelium, 0.8 uL/g [90], is only 0.4% of the brain ECS volume, 200 uL/g [60]. The rates of polymeric nanoparticle (PNP) endocytosis and exocytosis across a monolayer of mouse bEnd.3 endothelium in vitro have been estimated with a mathematical model [91]. The rates of nanoparticle endocytosis and exocytosis in an in vitro BBB model are much slower than the rates of antibody endocytosis and exocytosis at the BBB in vivo (Table 1). These differences may relate to the lack of receptor specificity of the PNPs, and the downregulation of the RMT process in cell culture [39].

## 4. Plasma Pharmacokinetics and the Brain Uptake of a High- and Low-Affinity TfRMAb

Once the in vivo rate constants of receptor endocytosis, exocytosis, and recycling are known, the TfRMAb mathematical model can be used to predict the brain concentration of a TfRMAb, or a TfRMAb fusion protein, at any time after IV administration of a given ID [35]. The brain concentration, relative to time, is used to compute the brain area under the concentration curve (AUC) using the trapezoid rule [35]. Based on the K_D_ of binding of the TfRMAb to the Tf-TfR complex, the rate constants of antibody dissociation from the Tf-TfR complex can be computed for a given rate constant of association (k_on_), e.g., 10^6^ M^−1^sec^−1^ (0.06 nM^−1^min^−1^) [35]. The corresponding rate constant of dissociation (k_off_) of the TfRMAb from the Tf-TfR complex is computed from k_off_ = k_on_·K_D_, where K_D_ is 0.36–3.6 nM for a high-affinity TfRMAb, is 36 nM for a moderate-affinity TfRMAb, or is 360 nM for a low-affinity TfRMAb [35]. The brain AUC values for TfRMAbs of varying affinity have been reported previously for an antibody with a k_on_ of 10^6^ M^−1^sec^−1^ or 10^5^ M^−1^sec^−1^ [35]. However, the brain AUC is also determined by the input function, which is the plasma AUC. The plasma AUC of a TfRMAb or HIRMAb may be strongly dependent on the properties of the therapeutic domain that is fused to the transporting antibody. The pharmacokinetic (PK) parameters of plasma clearance of a TfRMAb, a HIRMAb, or a HIRMAb-IDUA fusion protein have been determined in the Rhesus monkey at an ID of 3 or 30 mg/kg [24,92,93], and are shown in Table 2.

These PK studies show that the rate of plasma clearance of the model TfRMAb [24] and model HIRMAb [92] used in these modeling studies are comparable at an ID of 3 or 30 mg/kg administered by IV infusion (Table 2). However, when the lysosomal enzyme, IDUA, is fused to the HIRMAb, there is a large difference in the plasma clearance and plasma AUC of the HIRMAb-IDUA fusion protein [93], relative to the HIRMAb [92] (Table 2). The Cmax (A_0_) and plasma AUC values shown in Table 2 were originally reported in units of μg/mL and μg·min/mL, respectively [24,92,93]. These units were converted to nM, for A_0_, and to pmol·min/mL, for plasma AUC, based on a molecular weight of the TfRMAb or HIRMAb of 150 kDa, or a molecular weight of the HIRMAb-IDUA fusion protein of 300 kDa. The Cmax, or A_0_, of the HIRMAb-IDUA fusion protein is only 3–4% of the Cmax of the TfRMAb or HIRMAb (Table 2). The plasma AUC of the HIRMAb-IDUA fusion protein is only ~0.5–1% of the plasma AUC of the TfRMAb or HIRMAb (Table 2). The rate constant of plasma clearance, α, of the HIRMAb-IDUA fusion protein is only 5-13-fold greater than the α of plasma clearance of the TfRMAb or HIRMAb (Table 2), because α = CL/Vss, where CL = plasma clearance and Vss = systemic volume of distribution. The Vss of the HIRMAb-IDUA fusion protein is much higher than the Vss of the HIRMAb or TfRMAb alone. The Vss of the HIRMAb-IDUA fusion protein is 9–19-fold greater than the Vss of the TfRMAb or HIRMAb at an ID of 3 mg/kg, and is 5–7-fold greater than the Vss of the TfRMAb or HIRMAb at an ID of 30 mg/kg [24,92,93].

The high rate of plasma clearance of the HIRMAb-IDUA fusion protein, or the TfRMAb-IDUA fusion protein, compared to the HIRMAb or TfRMAb alone, is due to the mannose 6-phosphate (M6P) moieties on the IDUA domain of the fusion protein, and to the high-affinity binding of mannose 6-phosphorylated lysosomal enzymes to the cation-independent (CI) M6P receptor (M6PR) [94]. This M6PR is abundantly expressed in peripheral tissues but is not expressed at the BBB [36]. Consequently, the IDUA domain stimulates uptake of the IgG-IDUA fusion protein by peripheral organs but has no effect on the BBB transport of the fusion protein [36]. However, the IDUA domain indirectly has an important effect on the brain AUC of the IgG-IDUA owing to the marked reduction of the plasma AUC of the TfRMAb or HIRMAb following fusion of IDUA to the antibody (Table 2).

**Figure 8:**
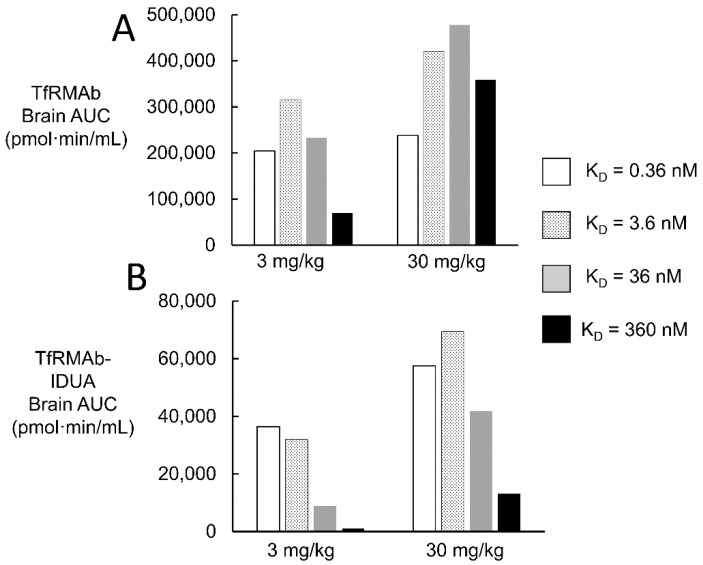
Brain AUC for a TfRMAb alone (**A**) and for a TfRMAb-IDUA fusion protein (**B**). Brain AUC is shown for an antibody with high affinity (K_D_ = 0.36–3.6 nM), moderate affinity (K_D_ = 36 nM), and low affinity (K_D_ = 360 nM). The association rate constant (k_on_) is the same, 10^6^ M^−1^sec^−1^, for all antibodies. Brain AUC was computed over 48 h after a single IV administration of either 3 or 30 mg/kg.

The impact of fusion of the IDUA enzyme to the TfRMAb on brain uptake was examined by modeling the brain concentrations over time of the TfRMAb alone, or the TfRMAb-IDUA fusion protein, and brain AUC, at an ID = 3 or 30 mg/kg, was computed. The plasma AUC, at a given time after injection, was determined from the A_0_ and α values for an ID of either 3 or 30 mg/kg of the TfRMAb (Table 2). The brain AUC for the TfRMAb alone was computed with the trapezoid rule as reported previously [35], and was computed for a TfRMAb with high-affinity, moderate-affinity, or low-affinity binding to the TfR, based on the dissociation constant (K_D_) of antibody binding to the TfR. The K_D_ varied over 3 log orders of magnitude, and ranged from K_D_ = 0.36–3.6 nM for a high-affinity TfRMAb, to K_D_ = 36 nM for a moderate-affinity TfRMAb, to K_D_ = 360 nM for a low-affinity TfRMAb. The brain AUC was then computed for a TfRMAb-IDUA fusion protein. The A_0_ and α value for the TfRMAb-IDUA fusion protein was assumed to be comparable to these values for the HIRMAb-IDUA fusion protein, owing to the comparable clearance of the TfRMAb alone or the HIRMAb alone in the primate (Table 2). The A_0_ value for the TfRMAb-IDUA fusion protein at an ID of 3 and 30 mg/kg is 15 and 340 nM, respectively, whereas the α value for the TfRMAb-IDUA fusion protein at an ID of 3 and 30 mg/kg is 0.010 and 0.014 min^−1^, respectively (Table 2). The brain AUC was determined for the period of 2880 min after IV infusion. The brain AUC for the TfRMAb alone, and for the TfRMAb-IDUA fusion protein, were computed for an ID of either 3 or 30 mg/kg, and the brain AUC values are shown in Figure 8A,B, respectively.

Comparison of the brain AUC for the TfRMAb alone (Figure 8A) with the brain AUC for the TfRMAb-IDUA fusion protein (Figure 8B) shows there is a 76–98% reduction in brain AUC when the IDUA is fused to the TfRMAb, relative to the TfRMAb alone, which parallels the marked reductions in plasma AUC of the TfRMAb-IDUA fusion protein, as compared to the TfRMAb alone (Table 2). The lower the affinity of the TfRMAb for the TfR, i.e., higher the K_D_, the greater reduction in the brain AUC caused by the reduced plasma AUC associated with the IDUA fusion protein. The brain AUC is increased 1.2-, 1.3-, 2.1-, and 5.1-fold as the ID is increased from 3 to 30 mg/kg, for the TfRMAb alone with a K_D_ of 0.36, 3.6, 36, and 360 nM, respectively (Figure 8A). The brain AUC is increased 1.6-, 2.2-, 4.7-, and 11.9-fold as the ID is increased from 3 to 30 mg/kg, for the TfRMAb-IDUA fusion protein with a K_D_ of 0.36, 3.6, 36, and 360 nM, respectively (Figure 8B). At a TfRMAb-IDUA fusion protein ID of 3 mg/kg, the brain AUC is directly related to the antibody affinity for the TfR (inversely related to K_D_) (Figure 8B). The brain AUC of the TfRMAb-IDUA fusion protein of moderate affinity, K_D_ = 36 nM, is 41,800 pmol·min/mL at an ID of 30 mg/kg (Figure 8B). Conversely, the brain AUC of the TfRMAb-IDUA fusion protein of high affinity, K_D_ = 0.36–3.6 nM, is 32,000–36,400 pmol·min/mL at an ID that is 10-fold lower, 3 mg/kg (Figure 8B).

## 5. Conclusions

Fitting mathematical models for the BBB receptor-mediated transcytosis (RMT) of either a TfRMAb, via the TfR, as illustrated in Figure 4, or a IRMAb via the IR, as illustrated in Figure 6, to experimental measurements of the brain uptake of a TfRMAb [24] or a HIRMAb-IDUA fusion protein [36] allows for estimates of the kinetics of the separate steps in the overall RMT process at the BBB (Table 1). These steps include receptor-MAb endocytosis into the brain capillary endothelium from plasma, exocytosis of the MAb into brain ECS from the intra-endothelial compartment, and receptor recycling back to the endothelial luminal membrane (Table 1). 

These modeling studies assume the K_D_ of antibody binding to the Rhesus monkey TfR or IR is comparable to the K_D_ of antibody binding measured for the human receptor ECD reported previously for the TfR or IR [35]. This assumption is supported by the 95–100% conservation of the amino acid sequence between humans and primates for the antibody epitope within the TfR [66] and IR [70]. However, even if the in vitro kinetics of antibody binding to the ECD of the Rhesus monkey TfR or IR was known, there would still be uncertainty as to whether such in vitro measurements made with the receptor ECD were operative at the receptor expressed within the endothelial luminal membrane at 37 °C in vivo. The TfR embedded within the endothelial membrane in vivo at 37 °C is a hetero-tetrameric complex of a TfR dimer and 2 holo-Tf molecules (Figure 1A), which is distinct from the in vitro condition of MAb binding at 23 °C to a single TfR ECD unbound by holo-Tf. A novel approach to the estimation of the in vivo kinetics of antibody binding to the endothelial TfR-Tf complex, or the endothelial IR, is afforded with the present methodology. The fitting of the TfRMAb or IRMAb models to the brain uptake data allows for in vivo estimation of the rate constant of MAb association with the endothelial receptor. The model fitting result for the TfRMAb shows the effective k_on_ of TfRMAb association with the Tf-TfR complex is 0.06 nM^−1^min^−1^, which is equal to 10^6^ M^−1^sec^−1^ (Figure 5). The model cannot fit the in vivo brain uptake data with a lower k_on_, 10^5^ M^−1^sec^−1^ (simulation 10, Figure 5). With regard to the association rate constant of IRMAb binding to the IR, the effective k_on_ in vivo is 10^5^ M^−1^sec^−1^ (simulation 15, Figure 7), as a k_on_ value of 10^4^ M^−1^sec^−1^ (simulation 19, Figure 7) or a k_on_ of 10^6^ M^−1^sec^−1^ (simulation 20, Figure 7) produces a predicted brain uptake that is either too low, or too high, respectively, relative to the experimentally observed brain uptake. These estimates of the k_on_ parameter are made in vivo at 37 °C and reflect binding of the MAb to the endothelial membrane-bound receptor, which is expressed at a receptor density, 0.03 fmol/mm^2^ (35), that is 100-fold lower than the receptor density used in in vitro SPR experiments [76,77].

Mathematical models for the transport of the endogenous ligands, holo-Tf (Figure 2) or INS (Figure 3), provide estimates of the concentrations of the unbound ligand, the ligand/receptor complex, or the unbound receptor within the brain capillary endothelium. The concentration in these pools shown in Figure 2 for holo-Tf transport, and shown in Figure 3 for INS transport, reflect the 5-log order of magnitude difference in the plasma concentration of holo-Tf, 25,000 nM, vs. the plasma concentration of INS, 0.3 nM [35]. The plasma holo-Tf concentration is nearly 1000-fold *greater* than the brain endothelial TfR concentration, 40 nM [35], whereas the plasma INS concentration is 100-fold *lower* than the brain endothelial IR concentration, 24 nM [35]. Consequently, only 5% of the total endothelial TfR is estimated to reside within the endothelial luminal membrane, and >99% of this luminal receptor is the Tf-TfR complex; the remaining 95% of endothelial TfR resides in the intra-endothelial compartment either as the free TfR recycling back to the membrane, or as a complex with holo-Tf (Figure 2). Conversely, 88% of the endothelial IR resides at the endothelial luminal membrane, and 95% of the luminal IR is in the form of the unoccupied IR, and not the INS-IR complex (Figure 3). Given these considerations, it is important to measure the affinity of a TfRMAb, which is being evaluated as a BBB delivery vector, for the complex of holo-Tf and the TfR, in addition to the free TfR. This is because binding of holo-Tf to the TfR induces conformational changes in the apical domain of the receptor [37,95], and most TfRMAbs, which do not inhibit holo-Tf binding to the TfR, bind to the apical domain of the receptor.

The model TfRMAb tested in these studies is a high-affinity bivalent antibody, which binds the TfR with a K_D_ value of 0.4 nM [24]. Similarly, pabinafusp alfa is a high-affinity bivalent TfRMAb [4,25]. TfRMAbs, such as those used to engineer the RO7126209 fusion protein [7] or the DNL310 fusion protein [6], are monovalent antibodies of either moderate- or low-affinity binding to the TfR, respectively. Monovalent TfRMAbs are said to be preferred delivery antibodies as it is believed that bivalent TfRMAbs cause TfR clustering within the cell, which leads to selective triage of the antibody to the lysosome and loss of TfR on the cell membrane [6,7]. However, this hypothesis that high-affinity bivalent TfRMAbs cause clustering of the TfR within the brain endothelial cell, followed by TfR degradation, is based on tissue culture experiments. Intracellular TfR clustering was induced in cultured malignant hematopoietic cell lines after cell exposure to a TfRMAb-avidin fusion protein, and the intracellular receptor clustering was attributed to the multivalency of the TfRMAb-avidin fusion protein [96,97]. However, an IgG-avidin fusion protein forms a tetravalent 400 kDa complex [96], due to the association of avidin monomers into tetrameric structures. The proapoptotic effect of the TfRMAb-avidin fusion protein was not observed with the bivalent TfRMAb not fused to avidin [96]. Apart from the polyvalency of IgG-avidin fusion proteins, there is no evidence that cell culture experiments showing TfRMAb-mediated intracellular TfR sequestration are relevant to in vivo transport at the BBB. In vivo investigations show no downregulation of the BBB TfR following chronic administration of a TfRMAb fusion protein. Mice were treated with 2 mg/kg administered IV twice weekly for 12 weeks with a fusion protein of a mouse-specific high-affinity bivalent TfRMAb and glial-derived neurotrophic factor [98]. At the end of 12 weeks of treatment, the BBB permeability-surface area (PS) product, which is a measure of TfR expression at the brain endothelial luminal membrane, was unchanged relative to the PS product measured without chronic treatment [98]. Chronic in vivo administration to mice of a high-affinity bivalent TfRMAb at a dose of 3 mg/kg causes no downregulation of brain TfR or brain iron [99]. If bivalent TfRMAbs were subject to sequestration within the capillary endothelium in vivo, then the TfRMAb exocytosis into the brain ECS would be impaired, and this would be reflected by in vivo measurements of brain antibody uptake. However, this is not observed in vivo with the model analysis of the brain uptake of a high-affinity bivalent TfRMAb (Figure 5). Simulations 2–5 in Figure 5 show that the predicted TfRMAb concentration in the brain ECS is progressively decreased from what is experimentally observed if the rate of exocytosis is reduced by 2-fold (simulation 2), 4-fold (simulation 3), or 6-fold (simulation 4). If exocytosis is eliminated, then there is a 100% reduction in the TfRMAb concentration in the brain ECS relative to what is experimentally observed in the primate brain in vivo (simulation 5, Figure 5). These modeling studies show a high-affinity bivalent TfRMAb moves rapidly and freely through the brain endothelial compartment to enter the brain ECS, confirming early in vivo work performed with internal carotid artery infusions of holo-Tf or a high-affinity bivalent TfRMAb [85]. 

The high-affinity bivalent TfRMAb or HIRMAb studied in the simulations shown in Figure 5 and Figure 7 are characterized by a T_1/2_ of dissociation from the TfR or IR of 31 and 120 min, respectively. These rates of dissociation approximate the rates of TfRMAb or HIRMAb exocytosis into the brain ECS, which occurs with a T_1/2_ of 5 and 20 min, respectively (Table 1). If an ultra-high-affinity TfRMAb or HIRMAb was developed that exhibited a log order lower rate constant of dissociation, e.g., MAb-receptor dissociation T_1/2_ >24 h, then the concentration of the MAb-receptor complex within the intra-endothelial compartment might increase to high levels, which would restrict the recycling of unoccupied receptor back to the luminal endothelial membrane. In this setting, the reduced concentration of the free IR or the Tf-TfR complex at the endothelial luminal membrane would be expected to cause diminished brain uptake of circulating INS or holo-Tf, respectively. This anomaly would be exacerbated by the administration of a high ID, e.g., 30 mg/kg, of the ultra-high-affinity TfRMAb or IRMAb. Although, as discussed below, the effects of a high ID are mitigated, in part, if the plasma clearance of the MAb fusion protein is rapid as exemplified by the IgG-IDUA fusion protein. The optimal receptor-binding properties would be a MAb with a K_D_ = 0.5–5 nM and an association rate constant (k_on_) of 10^5^–10^6^ M^−1^sec^−1^, which would produce a dissociation T_1/2_ of ~10–120 min. A targeting MAb with these kinetic properties enables a therapeutic brain delivery at a low injection dose of 3 mg/kg (Figure 8). Either a TfRMAb or a IRMAb with these properties would provide a comparable level of brain delivery.

The brain uptake of a TfRMAb or IRMAb, as reflected in the brain AUC (Figure 8), is a function not only of the antibody affinity for the receptor and the injection dose, but also is controlled by the plasma AUC, which is the input function for antibody delivery to the brain. The biologic fused to the TfRMAb or IRMAb may have a marked effect on the antibody plasma AUC, as illustrated for an IDUA fusion protein (Table 2). The rapid plasma clearance of the IgG-IDUA fusion protein is due to the high affinity of IDUA for the CI M6PR [91]. Fusion of IDUA to the TfRMAb causes a reduction in the brain AUC of the fusion protein compared to the TfRMAb alone, and the effect of the IDUA fusion is inversely related to receptor affinity, i.e., directly related to K_D_ (Figure 8B). At an ID of 3 mg/kg, the brain AUC of the TfRMAb-IDUA fusion protein is reduced 6-fold, 10-fold, 26-fold, and 64-fold, compared to the brain AUC of the TfRMAb alone, when the KD is 0.36, 3.6, 36, and 360 nM, respectively (Figure 8). The lower the affinity of the antibody for the TfR, the greater the ID required to maintain a given brain AUC. For example, the brain AUC of a TfRMAb-IDUA fusion protein with a moderate affinity for the TfR, KD = 36 nM, at an ID = 30 mg/kg, is comparable to the brain AUC of a TfRMAb-IDUA fusion protein, KD = 0.36–3.6 nM, at a 10-fold lower ID of 3 mg/kg (Figure 8B). 

The lower the affinity of the TfRMAb for the TfR, the higher the ID required to produce a brain AUC comparable to that generated with a high-affinity TfRMAb [35]. This effect of reduced affinity is augmented when the fusion partner, e.g., IDUA, causes an accelerated plasma clearance of the antibody. High IDs, e.g., 30 mg/kg, as compared to 3 mg/kg, can reduce the therapeutic index of the IgG fusion protein. Toxicity could arise from either the IgG domain or the therapeutic domain of the fusion protein following the administration of high doses, such as 30 mg/kg. Conversely, the use of a high-affinity antibody, e.g., KD = 0.36–3.6 nM, allows for an adequate brain AUC at a lower injection dose of 3 mg/kg.

These modeling studies were performed with experimental data derived from the healthy Rhesus monkey [24,36]. In disease states, any of the intermediate steps involved in the RMT process shown in Figure 4 and Figure 6 could be altered. Transcytosis would be impaired if endothelial dysfunction in a diseases state affected endocytosis at the luminal membrane, exocytosis at the abluminal membrane, or intracellular receptor recycling. For the TfRMAb model, simulations 1–5 and simulations 6–9 show the effect of impaired exocytosis and endocytosis, respectively (Figure 5). For the IRMAb model, simulations 13–17 and simulations 11–13 and 18 show the effect of impaired exocytosis and endocytosis, respectively (Figure 7). Endothelial dysfunction impacting on the RMT process could take place in different CNS diseases, including neurodegeneration, vasculitis, brain tumors, or stroke.

## Figures and Tables

**Table 1:** Mathematical model estimates of BBB transcytosis via the TfR and IR.

Transport Component	TfR	IR
T_1/2_ of receptor endocytosis	5–10 min	30 min
T_1/2_ of MAb exocytosis	5 min	20 min
T_1/2_ of receptor recycling	20 min	20 min
k_on_ of MAb binding to receptor	10^6^ M^−1^sec^−1^	10^5^ M^−1^sec^−1^
Plasma endogenous ligand	holo-Tf = 25,000 nM	insulin = 0.3 nM
Total endothelial receptor	TfR = 40 nM	IR = 24 nM
Luminal endothelial receptor	TfR = 2 nM	IR = 21 nM

**Table 2:** Pharmacokinetic parameters of plasma clearance of a TfRMAb, HIRMAb, and HIRMAb-IDUA fusion protein in the Rhesus monkey after IV administration of 3 or 30 mg/kg.

PK Parameter	ID (mg/kg)	TfRMAb [24]	HIRMAb [92]	HIRMAb-IDUA [93]
A_0_ (nM)	3	373 ± 80	473 ± 74	15 ± 3
α (min^−1^)		0.0021 ± 0.0002	0.00077 ± 0.00018	0.010 ± 0.002
plasma AUC		100,473 ± 7920	593,126 ± 135,006	1190 ± 146
A_0_ (nM)	30	3240 ± 120	5446 ± 113	343 ± 18
α (min^−1^)		0.0010 ± 0.0001	0.0011 ± 0.0001	0.014 ± 0.004
plasma AUC		3,187,113 ± 326,420	4,291,786 ± 581,686	25,243 ± 5426

Units of plasma AUC are pmol·min/mL; A_0_ = Cmax; ID = injection dose.

## Data Availability

Data sharing not applicable.

## Abbreviations

α: rate constant of MAb clearance from plasma; AUC, area under the plasma or brain concentration curve; BBB, blood-brain barrier; BHK, baby hamster kidney; CBF, cerebral blood flow; CI, cation independent; Cmax (A_0_), maxima lplasma concentration; CT, carboxyl terminus; ECD, extracellular domain; ECS, extracellular space; HIR, human insulinreceptor; HIRMAb, MAb against HIR; HIRMAb-IDUA, fusion protein of HIRMAb and IDUA; HIRMAb-IR, complex of HIRMAb-IDUA fusion protein and IR; ID, injection dose; IDS, iduronate 2-sulfatase; IDUA, α-L-iduronidase; IGF, insulin-like growth factor; IGFR, IGF receptor;I NS, insulin; INS-IR, complex of INS and IR; IR, insulin receptor; IR-A, short form of IR; IR-B, long form of IR; IRMAb, MAb against IR; IV, intravenous; k_CBF_, rate constant of cerebral blood flow; K_D_, dissociation constant; k_endo_, rate constant of endocytosis; k_exo_, rate constant of exocytosis; k_off_, rate constant of dissociation; k_on_, rate constant of association; LEPR, leptin receptor; M6P, mannose 6-phosphate; M6PR, M6P receptor; MAb, monoclonal antibody; MAb-Tf-TfR, complex of TfRMAb, Tf, and TfR; MPS, Mucopolysaccharidosis; MPSI, Type I MPS; MPSII, Type II MPS; NAR, new antigen receptor; PNP, polymeric nanoparticle; PS, permeability-surface area; RMT, receptor-mediated transcytosis; ScFv, single chain Fv antibody; SPR, surface plasmon resonance; T_1/2_, half-time; Tf, transferrin; TfR, transferrin receptor; TfRMAb, MAb against TfR; TfRMAb-IDS, fusion protein of TfRMAb and IDS; Tf-TfR, complex of Tf and TfR; VNAR, variable domain of NAR IgG; Vp, brain plasma volume.
